# Lipidomics unveils the complexity of the lipidome in metabolic diseases

**DOI:** 10.1186/s40169-018-0182-9

**Published:** 2018-01-26

**Authors:** Todd A. Lydic, Young-Hwa Goo

**Affiliations:** 1 Department of Molecular and Cellular Physiology, Albany Medical College, 47 New Scotland Avenue, Albany, NY 12208 USA; 2 Department of Physiology, Michigan State University, East Lansing, MI 48824 USA

**Keywords:** Lipid, Lipidomics, Lipidome, Metabolism, Metabolic disease, Lipoprotein, Lipid droplet, Atherosclerosis, Mass spectrometry

## Abstract

Dysregulation of lipid metabolism is responsible for pathologies of human diseases including metabolic diseases. Recent advances in lipidomics analysis allow for the targeted and untargeted identification of lipid species and for their quantification in normal and diseased conditions. Herein, this review provides a brief introduction to lipidomics, highlights its application to characterize the lipidome at the cellular and physiological levels under different biological conditions, and discusses the potential for the use of lipidomics in the discovery of biomarkers.

## Introduction

Lipids are hydrophobic or amphipathic molecules that contain unique structural and biological properties. They originate entirely or in part from biochemical building blocks of ketoacyl thioesters and/or isoprenes [1, 2]. Lipids function as an energy reservoir and serve as major structural components of biological membranes such as plasma membranes, membranes of intracellular organelles, and lipid transporters [3]. Lipids also participate in signal transduction as chemical messengers and interact with proteins to regulate their functions [4, 5]. Furthermore, lipids are important bioactive molecules in the body’s immune system against viral and bacterial infections [6].

Metabolomics is the systemic study of metabolites generated during biological processes in cells, tissues, and organisms. A Medline search on the PubMed website shows a renaissance of metabolomics research in recent years driven by the advancement of multiple technology platforms such as mass spectrometry (MS). Unlike the global studies of DNA (genomics), RNA (transcriptomics), and protein (proteomics) that are well established, progress in developing global lipidomics has more recently accelerated due to dramatic improvements in MS technology together with bioinformatics. Like other “omics”, lipidomics facilitates systemic lipid profiling from cells or biological fluids, tissues, or even whole organisms. Thus, the emergence of lipidomics as a mature sub-field of metabolomics now enables a complete characterization of the metabolome, at least in theory. This will greatly contribute to the development of diagnostic tools, drugs, and therapeutic strategies for fighting metabolic diseases. This review briefly introduces lipidomics, describes lipid distribution at the cellular level as well as in circulation, and highlights recent discoveries related to the lipidome and biomarkers driven by lipidomics analysis at the cellular and physiological level.

## Lipid classes

In 2005, the LIPID MAPS consortium, the International Lipid Classification, and National Nomenclature Committee grouped lipids into eight categories based on chemical and biochemical principles of lipids: fatty acyls, glycerolipids, glycerophospholipids, sphingolipids, sterol lipids, prenol lipids, saccharolipids, and polyketides (Table 1) [1, 7]. Each category of lipid is further divided into subclasses that have diverse structural and chemical properties. Since lipids are broadly defined as any molecule that is insoluble in water or soluble in organic solvents, a variety of molecules belong to these broad lipid categories. As of January 2018, more than 40,000 lipid structures are listed in the LIPID MAPS database (http://www.lipidmaps.org). Due to rapid and continuing improvements in lipid analytical tools, more lipid species are certain to be identified in the near future.

**Table 1:** Lipid categories, their classes and the structure of representative of each lipid category

Category	Class
Fatty acyls [FA]	Fatty acids and conjugates Octadecnoids Eicosanoids Docosanoids Fatty alcohols Fatty aldehydes Fatty esters Fatty amides Fatty nitriles Fatty ethers Hydrocarbons Oxygenated hydrocarbons Fatty acyl glycosides Other fatty acyls
Glycerolipids [GL]	Monoradylglycerols Diradylglycerols Triradylglycerols Glycosylmonoradylglycerols Glycosyldiradylglycerols Other glycolipids
Glycerophospholipids [GP]	Glycerophosphocholines Glycerophosphoethanolamines Glycerophosphoserines Glycerophosphoglycerols Glycerophosphoglycerophosphates Glycerophosphoinositols Glycerophosphoinositol monophosphates Glycerophosphoinositol bisphosphates Glycerophosphoinositol trisphosphates Glycerophosphates Glyceropyrophosphates Glycerophosphoglycerophosphoglycerols CDP-glycerols Glycosylglycerophospholipids Glycerophosphoinositolglycans Glycerophosphonocholines Glycerophosphonoethanolamines Di-glycerol tetraether phospholipids (caldarchaeols) Glycerol-nonitol tetraether phospholipids Oxidized glycerophospholipids Other glycerophospholipids
Sphingolipids [SP]	Sphingoid bases Ceramides Phosphosphingolipids Phosphonosphingolipids Neutral glycosphingolipids Acidic glycosphingolipids Basic glycosphingolipids Amphoteric glycosphingolipids Arsenosphingolipids Other sphingolipids
Sterol Lipids [ST]	Sterols Steroids Secosteroids Bile acids and derivatives Steroid conjugates Other sterol lipids
Prenol Lipids [PR]	Isoprenoids Quinones and hydroquinones Polyprenols Hopanoids Other prenol lipids
Saccharolipids [SL]	Acylaminosugars Acylaminosugar glycans Acyltrehaloses Acyltrehalose glycans Other acyl sugars Other saccharolipids
Polyketides [PK]	Linear polyketides Halogenated acetogenins Annonaceae acetogenins Macrolides and lactone polyketides Ansamycins and related polyketides Polyenes Linear tetracyclines Angucyclines Polyether antibiotics Aflatoxins and related substances Cytochalasins Flavonoids Aromatic polyketides Non-ribosomal peptide/polyketide hybrids Phenolic lipids Other polyketides

### Identification of lipids by lipidomics

**Fig. 1:**
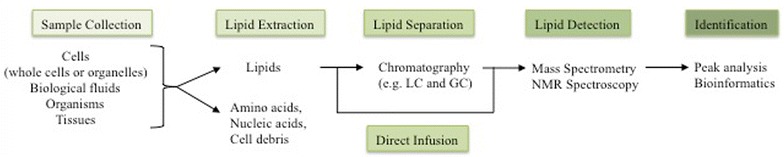
Workflows of lipidomics. Lipids are extracted in organic solvents from various samples. Extracted lipids are either further separated by a chromatography or directly infused to mass-spectrometry. Peak analysis identified lipid species

Lipidomics is a subset of metabolomics that aims to identify and quantify a large number of lipids at the same time. To achieve this, a lipidomics analysis involves multiple processes: extraction of lipids from a biological sample, separation, detection, and data analysis (Fig. 1). The method to be applied at each step is dependent upon the chemical and biochemical characteristics of the analytes of interest, as well as the abundance of each target lipid.

### Lipid extraction and separation

Prior to detection, lipids are isolated from nucleic acids and proteins using organic solvents. Most lipids can be extracted by the Folch method or the Bligh and Dyer procedure. Both methods partition lipids into an organic phase by using different ratios of chloroform/methanol solvents: Folch method (chloroform:methanol = 2:1) and Bligh and Dyer method (chloroform:methanol = 1:2) [8, 9]. Depending on the hydrophobicity and the abundance of lipids, several alternative solvents such as hexane, isopropanol, ethyl acetate, and tert-butyl methyl ether can be used with similar extraction efficiency for major lipid classes but with varying effectiveness for the recovery of low abundance lipid classes [10]. In order to avoid the loss of more polar lipid species to partial or full partitioning to the aqueous phase during biphasic lipid extraction methods, monophasic lipid extraction methods have been developed to promote full recovery of a broader range of lipid classes, thus facilitating more comprehensive lipidomics analyses. Monophasic extraction strategies may also simultaneously facilitate recovery of polar metabolites to enable analysis of the entire metabolome. Monophasic extraction methods include variants of the methanol/chloroform/water mixtures discussed above or methanol, acetonitrile, and acetone [11, 12]. After extraction, the lipids are often separated by various chromatographic methods such as thin layer chromatography (TLC), gas chromatography (GC), and high-performance liquid chromatography (HPLC), or lipids can be directly infused to a mass spectrometer for detection without separation.

### Lipid detection

For lipidomics analyses, lipids are detected by MS or non-MS based methods [13, 14]. Due to its high sensitivity and possibility for performing high throughput analyses, MS has recently become the most popular detection method of choice [15]. Chromatography associated with MS (e.g., LC/MS) is optimized for the detection and separation of specific lipid classes that are structurally similar, and it provides superb analytical specificity for each lipid species in a given class [11]. However, given that lipids are diverse in polarity and abundance, chromatographic separation often precludes obtaining truly global lipidome profiling in a single analytical run. In addition, chromatographic separation is often time intensive and generates high error rates due to multiple steps involved in the process and requires a large amount of sample [11, 16]. By contrast, shotgun lipidomics directly introduces lipids that were ionized by the soft electrospray ionization (ESI), the matrix assisted laser desorption/ionization (MALDI), or desorption electrospray ionization (DESI), and detects the lipids by MS, with separation of lipids achieved solely on the basis of mass-to-charge ratio. This enables the resolution of each lipid class without chromatographic separation and reduces analysis time as well [16–18]. Thus, shotgun lipidomics allows untargeted high-throughput lipid profiling from a limited amount of biological sample, despite the presence of complex mixtures of various lipid classes. However, direct infusion or ‘shotgun’ lipidomics approaches cannot distinguish isobaric lipid species within the same class in the crude extract, and the numbers of lipid species that can be detected by this analysis remains at several hundred, compared to 10,000–100,000 of the predicted eukaryotic lipid species [3, 19–21]. Therefore, chromatography-based lipidomics and shotgun lipidomics can be considered as complementary approaches depending on the purpose of lipidomics analysis, such as whether a targeted or untargeted analysis is required. Nuclear magnetic resonance spectroscopy (NMR), a non-MS based lipidomics platform, is a noninvasive method that enables detection and quantification of metabolites including lipids [22, 23]. However, its modest sensitivity and limited resolving power compared to MS limits its applications for lipidomics analyses [24]. Lately, NMR has gained attention due to its ability to accurately quantify circulating lipoprotein particles and lipids, as well as other metabolic parameters [15, 25]. This study also demonstrated the feasibility of NMR for high throughput screening of lipids in human plasma, which can be used in clinics as a diagnostic tool.

### Lipid quantification

In MS analysis, lipid species are identified by mass and/or MS/MS spectral features, and the abundances of individual lipid species may be quantified by ratiometric comparison of peak intensity or peak area against one or more internal standards. Strategies for quantitation of lipid species in lipidomic analyses vary based on the analytical approach. For targeted multiple reaction monitoring (MRM) based identification of a small number of lipid species, it may be feasible to include heavy isotope or otherwise chemically labeled exogenous lipids as internal standards for absolute quantitation. However, if the number of lipid species to be quantitated is sufficiently large during targeted MRM experiments, or if a global/untargeted analysis is required, it is untenable to include labeled internal standards for precise quantitation of each known or hypothetical lipid molecular species. Therefore, one strategy that has been employed for quantitation of lipid species across a large number of lipid classes is to include an exogenous lipid molecular species representative of each lipid class of interest. For example, Avanti Polar Lipids provides commercially available ‘cocktails’ of lipid standards such as the SPLASH^®^ Lipidomix^®^ Mass Spec Standards (http://www.avantilipids.com). This approach enables correction for differences in ionization efficiency of each lipid class owing to the inherent polarity of each lipid head group or backbone structure. However, it should be noted that the lipid head group is only one factor in determining the ionization efficiency of a lipid molecular species [26]. Additionally, it has been well established that the length and degree of unsaturation of the acyl chains esterified to the lipid backbone have a significant influence on the relative ionization efficiency of a given molecular species [26]. As it is not possible to provide a sufficient number of exogenous lipid standards to correct for differences in ionization efficiency for each unique combination of lipid head group and fatty acyl chains (of which there are thousands of hypothetical permutations), an alternative approach is to use only one exogenous lipid molecular species as a standard for relative quantitation and normalization of slight differences in ESI spray efficiency across samples [27]. In this case, data are typically reported as relative percent abundances, fold change, or as normalized ion abundances, without attempting to provide absolutely quantitative values. Regardless of which approach is used for quantitation of lipidomics datasets, any attempt to quantitatively apply electrospray ionization for mass spectrometry based lipid analysis must include an evaluation of ion suppression effects to determine whether the analytical conditions employed provide a detector response that changes linearly with sample concentration. Highly abundant analytes and/or sample matrix components may utilize the majority of the available charge in an electrospray droplet, leaving lower abundance analytes to be disproportionately uncharged, and therefore underdetected, during ESI-based MS analysis [26, 28]. The practical consequence of this effect is that samples containing lipids at high concentrations will exhibit suppression effects if samples are not adequately diluted prior to ESI-MS analysis. Therefore, evaluation of ion suppression effects as a function of sample dilution should be a critical step in lipidomics quantitation for both direct infusion and LC–MS approaches.

### Data analysis strategies

Evaluation of lipidomics datasets may performed manually, or by utilizing software platforms for peak identification and quantification of lipid species. Manual interpretation of data for either LC–MS or direct infusion requires inspection of MS peak abundances and/or peak area calculations, and comparison of MS and MS/MS mass-to-charge values and/or retention times to those of previously analyzed standards or publically available datasets. While the manual interpretation of direct infusion or LC–MS spectra may be feasible for relatively simple mixtures or a small number of samples, this approach becomes daunting when large datasets and/or complex mixtures of lipids, such as those obtained from biological samples, are characterized. Alternatively, numerous software platforms have been developed to simplify specific aspects of lipidomics data analysis, or to serve as seamless data analysis suites capable of performing peak identification, quantitation, and statistical analysis across large-scale datasets. At the simplest level, algorithms may be utilized for correction of ^13^C isotope effects to simplify lipidomics datasets prior to downstream manual inspection/data processing [29]. To facilitate streamlined interpretation of lipidomics data, any lipidomics data analysis tool should also minimally be capable of normalizing MS peak areas to one or more internal standard. Ideally, a data analysis platform should also include automated searches against metabolite databases such as lipid MAPS (http://www.lipidmaps.org), Lipid Bank (http://lipidbank.jp/), LipidHOME (http://www.ebi.ac.uk/metabolights/lipidhome) and LipidBlast (http://fiehnlab.ucdavis.edu/projects/lipidblast) [30, 31]. Freely available software which combines one or more of the above steps includes the Analysis of Lipid Experiments (ALEX), Lipostar, MS-DIAL, and lipid mass spectrum analysis (LIMSA) platforms [32–35]. Numerous commercially available and instrument vendor-specific software packages have also been developed to streamline lipidomics data analysis.

## Bioinformatics and pathway analysis

An important application of bioinformatics in metabolomics data mining is to facilitate pathway analysis [36, 37]. Lipidomics, especially global/untargeted lipidomics, has the power to reveal a broad snapshot of metabolic events occurring in both closely and distantly related pathways. However, deriving meaning from peak lists spanning dozens or hundreds of samples is no simple task. Manual interpretation of data using publically available databases such as KEGG pathways and the LipidMAPS databases may help place quantitated lipidomics results into a meaningful biological context. Similarly, an analyst may rely on known principles of lipid biochemistry to calculate indexes of fatty acid unsaturation, fatty acyl chain length, or fatty acid precursor/product ratios to gain insight into the function of fatty acid remodeling or other relevant lipid metabolic pathways. As with any data mining exercise, manual curating of data is inherently time-consuming and requires considerable knowledge of relevant pathways and principles. Extremely large datasets spanning hundreds of samples may simply preclude the feasibility of manual bioinformatics approaches altogether. Alternatively, bioinformatics software platforms may provide assistance with lipid pathway analysis, including some software packages that are available free of charge, such as VANTED and MAVEN [38, 39]. Such software can help place key findings of a lipidomics experiment into a simple, user-friendly readout of affected pathways and analytes, with considerable savings in the time required. Commercially available software packages are also available from numerous vendors, which may support or facilitate lipid pathway analysis.

## Applications of lipidomics

### Cellular lipids

**Fig. 2:**
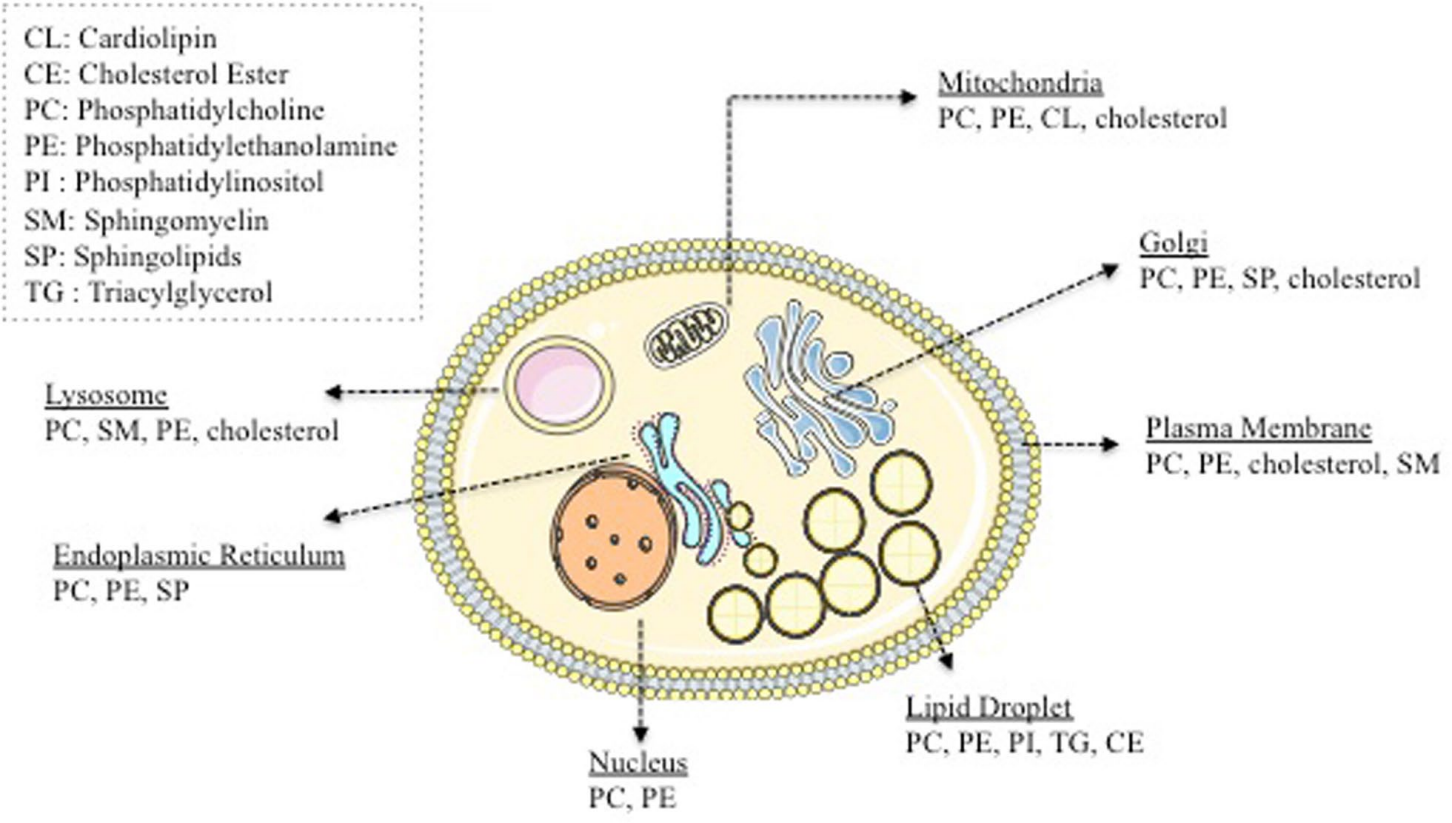
Lipid distribution of animal cells. In animal cells, lipids are distributed in the plasma membrane (PM) and intracellular organelles. In all membranes, most commonly found lipids are phosphatidylcholines (PC) followed by phosphatidylethanolamines (PE). Cardiolipin is prevalently found in mitochondria. Extra FAs and free cholesterols are esterified in the ER and stored within cytosolic lipid droplet (LD) as triacylglycerol (TG) and cholesterol ester (CE) respectively. Lipid rafts are concentrated with cholesterols and sphingolipids

Lipids in eukaryotic cells are distinctively distributed in diverse proportions, compositions, and concentrations in the membranes of intracellular organelles (Fig. 2) [40–42]. Furthermore, the lipidome of each organelle may be remodeled by extra- and intra-cellular stimulation, that can also affect lipid trafficking among the organelles [43]. In cells, phospholipids comprise the most abundant lipid class. Phosphatidylcholine (PC) and phosphatidylethanolamine (PE) are major subclasses of phospholipids found in the PM and intracellular organelles (Fig. 2) [40, 41]. The PM of mammalian cells functions as a barrier and is composed of a phospholipid bilayer with embedded proteins that function as receptors, transporters, or enzymes. Lipids of the PM occupy about 50–60% of all cellular lipids [43]. The major lipid constituents of the PM are phospholipids, sterols, and sphingolipids [41]. Phospholipids and glycerolipids in the PM are metabolized to bioactive lipid mediators that are responsible for downstream inflammatory responses. For example, after inflammatory activation, diacylglycerol and PC in the PM can release arachidonic acid (AA) by the action of phospholipase C and phospholipase A2, respectively. Liberated AA is converted into various species of prostaglandins and leukotrienes that are involved in inflammatory responses [44]. Lipid rafts are cholesterol and sphingolipid rich regions of the PM [45]. Phospholipids in lipid rafts contain more saturated fatty acyl chains than phospholipids in the non-lipid raft area of the PM. Saturated FAs in the raft sphingolipids and phospholipids are closely packed with cholesterol resulting in more ordered and less fluid membranes than the rest of the PM. Many signaling proteins are concentrated in lipid rafts via direct lipidation (e.g., palmitoylation) or lipid-binding domains, which promote the function of lipid rafts as signaling platforms [45, 46].

The endoplasmic reticulum (ER), where many lipogenic enzymes and hydrolases are located, is a major site of lipid biogenesis. Phospholipids, cholesterol, sphingolipids, and neutral lipids such as triacylglycerol (TG), as well as sterol esters, are synthesized in the ER and ultimately transported to other organelles [41]. While cholesterol is synthesized in the ER, less than 1% of total cholesterol is located in the ER [41, 47]. After synthesis, cholesterol is rapidly transported to the PM where 60–70% of all cellular cholesterol is found. Excess accumulation of the free forms of lipids such as free FA and free cholesterol in the ER causes the ER stress response, resulting in cellular apoptosis and necrosis [48, 49]. Therefore, the ER converts the surplus of cytotoxic free forms of lipids into neutral forms (e.g., TG and sterol esters), which in turn are stored in cytosolic lipid droplets (LDs) [4].

Lipid droplets consist of a monolayer of phospholipids and associated proteins. Metabolic cues (e.g., feeding and fasting) not only cause morphological changes to LDs such as fusion, expansion, and shrinking, but also change the lipid composition of LDs. During the growth of LDs in the cytosol, lipid synthesis also occurs at the LDs by relocation of lipid synthesizing enzymes to the LDs [50]. The Golgi apparatus is the central organelle for sorting and transporting proteins as well as lipids. The Golgi can also synthesize some lipid classes such as sphingolipids, and play a role in the sorting of membrane proteins and lipids [41, 51]. The lysosome is the primary site of lipid catabolism. Lysosomes contain ~ 60 hydrolases including many lipases [52]. Inherited mutations in lysosomal lipases cause lysosomal lipid storage diseases due to cellular lipid imbalance [53]. Although mitochondria import lipids from the ER, certain mitochondrial lipids such as cardiolipin and phosphatidylglycerol, as well as some PC and PE, are synthesized in the mitochondria [54]. Cardiolipin is exclusively found in the inner membrane of mitochondria, and cardiolipin and PE are important for cristae development and the respiratory function of mitochondria [40, 55].

### Cellular lipidomics

Lipidomics approaches utilizing whole cell lysates of cultured cell lines or primary cells support the notion that, like the transcriptome and proteome, the cellular lipidome is actively remolded under various stimuli and physiological conditions [56]. For example, fatty acid (FA) desaturation and elongation enzymes are strongly induced when monocytes, elutriated from healthy donors, differentiate into macrophages. The analysis of lipidome profiles during this process found that polyunsaturated FAs and monounsaturated FAs in the PE plasmalogens are decreased and increased, respectively, and the profiles are similar to those of circulating mature granulocytes [57]. Plasmalogens are important precursors of lipid mediators that regulate inflammation and phagocytic function of the macrophages, providing lipid profile as a marker of macrophage activation. In another lipidomics study of five different prostate cancer cell lines, 23 of 26 glycerophospholipids and SM (d18:1/16:0) were found to be elevated in metastatic cancer cells compared to normal prostate epithelial cells and non-malignant cells [58]. Among the glycerophospholipids, two types of PCs [PC (16:0/16:1) and PC (18:0/18:1)] were strongly increased in the metastatic cancer cells (> 31.4-fold and > 20.3-fold, respectively). Lipidomics analysis of inflammatory Toll-like receptor agonist treated Raw 264.7 cells, a murine macrophage cell line, found that Toll-like receptor activation induces an early response of FA metabolism shown by increased eicosanoid synthesis and remodeling of major lipid species including glycerolipids, glycerophospholipids, and prenols [20]. This study presented the system level of the cellular lipid profile in macrophages during inflammatory stimulation. Since lipidomics of total cell lysates necessarily disturbs the spatial distribution of all cellular lipids, organelle-specific lipidomics may be preferred to gain insight into lipid metabolism at the subcellular level. A comprehensive lipidomics analysis of subcellular organelles isolated from resting and activated macrophages found that during macrophage activation some changes in lipid composition occur in all organelles, whereas other changes are manifest only in specific organelles [59]. In other studies, lipidomics of only selected organelles was performed. A shotgun lipidomics study of isolated lysosomes revealed that the lysosomal lipidome is selectively changed in Niemann–Pick disease type C1 (NPCI) deficient cells even though the plasma membrane lipidome remained unchanged [60]. In this study, in addition to confirming the accumulation of cholesterol and glycosphingolipids which are the hallmark of NPC1 deficiency, lipidomics analysis also revealed the build-up of several glycerophospholipids and other storage lipids [60]. To study the spatial and temporal distribution of lipid species and their abundances, a shotgun lipidomics study of isolated nuclei and mitochondria of mouse liver was performed. In this study, the authors identified common and organelle-specific lipid species and also found that the mitochondrial lipidome exhibits more temporal fluctuation than the nuclear lipidome [42]. Lipidomics of the purified LD fraction of hepatocytes from high fat diet-fed and fasting mice showed that TG species, which are a major component of LD, exhibit diverse profiles dependent on the feeding condition [61, 62]. ER lipid profiling by a shotgun lipidomics in a *Mycobacterium tuberculosis* infected macrophage cell line revealed significant alterations of phospholipid composition [63]. These organelle-specific lipidomic analyses described above demonstrate that organelle function and lipid composition are intertwined, and change in parallel in response to different physiological conditions.

### Lipidomics of plasma

Daily physiological conditions such as fasting and feeding, genetic background, and disease conditions can alter plasma lipid concentrations and composition. In order to distribute lipids to tissues, plasma lipids are carried by lipoproteins in the body’s circulation. Lipoproteins consist of lipids and apoproteins [64]. Based on their densities and associated proteins, lipoproteins are classified into seven classes: chylomicron (CM), CM remnant, very low density lipoprotein (VLDL), low density lipoprotein (LDL), LDL remnant, lipoprotein(a) [Lp (a)], and high density lipoprotein (HDL) [64]. Phospholipids and non-esterified cholesterols make up the surface of lipoproteins along with multifunctional apolipoproteins, which stabilize the structure and determine the functionality of lipoproteins. The core of lipoproteins contains non-polar lipids, primarily TGs, and sterol esters. Each class of lipoprotein possesses a unique function in delivering lipids to tissues. Chylomicrons are synthesized in enterocytes and primarily carry TGs obtained from our daily diet [65]. The liver produces VLDL particles and releases them into circulation to deliver TGs. LDL carries mostly cholesterol and is generated when VLDL loses the majority of its TGs. Lp (a) is an LDL like a lipoprotein and is also enriched with cholesterol. Unlike LDL, Lp (a) is produced in the liver. Lipid composition of Lp (a) is similar to LDL but Lp (a) also contains apolipoprotein A, which is absent in LDL [66]. HDL is produced in the liver as a lipid-poor lipoprotein and brings cholesterol from peripheral tissues to the liver, where cholesterol is metabolized to bile acids and ultimately secreted [67]. Each lipoprotein exhibits discrete compositions and concentrations of lipids and apolipoproteins. Lipoproteins interact with each other and they exchange lipids and apolipoproteins [68]. Therefore, changes in the number of lipoprotein particles, lipid composition and concentration, apolipoprotein modification, or a defect in lipid exchange among lipoproteins reflect the functionality of lipoproteins and manifest signs of metabolic diseases. Over the last 50 years, simple lipid profiling to measure plasma cholesterol such as LDL-C and non-HDL-C has been widely used in clinics as a common metabolic parameter and a diagnostic tool for cardiovascular diseases (CVD) [68]. Human cohort studies support that cumulative exposure to higher levels of plasma LDL cholesterol is linked to a prevalent risk of atherosclerotic CVD [69]. However, this method does not explain the existence of substantial numbers of patients that developed CVD despite having a normal range of plasma cholesterols. This requires a more comprehensive lipid analysis such as evaluation of the particle number of lipoproteins, modification of apolipoproteins, and variability of lipid composition across individuals rather than assessment of simple LDL cholesterol levels although several factors such as inflammations, diabetes and other pre-existing conditions can affect the prevalence of CVD [10, 70, 71]. Indeed, comprehensive lipid analysis has identified those parameters can be utilized as additional parameters to distinguish CVD risk groups that have similar plasma LDL-C and non-HDL-C. In addition, it is known that human plasma lipids are much more complex than the sterols examined in the above lipid measurements. A comprehensive lipidomics study of total plasma from fasting, healthy individuals led to the identification of more than 500 lipid species that span six different categories of lipids, including fatty acyls, glycerolipids, glycerophospholipids, sphingolipids, sterols, and prenols [72]. These plasma lipids are distributed to each lipoprotein in different proportions and concentrations. Another lipidomics study of VLDL, LDL, and HDL separated by ultracentrifugation of healthy human plasma identified 157 lipid species in the plasma, 182 in LDL, 171 in HDL, and 148 in VLDL [72]. Furthermore, this study also found that most plasma sphingomyelins are dominantly found in LDL particles and that most plasma lysophosphatidylcholines are found in HDLs [71]. In neurons of the brain, 24-hydroxycholesterol is enzymatically synthesized from cholesterol by a neuron-specific enzyme, cytochrome P450 46A1 (CYP46A1) [73]. Due to the predominant expression of CYP46A1 in the cerebrum of the brain, detectable 24-hydroxycholesterol in circulation is exclusively derived from the brain, although CYP46A1 is also weakly expressed in the liver. In contrast to cholesterol, 24-hydroxycholesterol can cross the blood–brain barrier, and it is metabolized to bile acids in the liver. Therefore, this is considered to be a major route of cholesterol secretion from the brain to circulation. While total cholesterol levels are unchanged, 24-hydroxycholesterol is significantly higher in the serum of Alzheimer’s disease and vascular dementia patients compared to healthy individuals [74].

### Remodeling of the lipidome in disease

It is clear that the lipidome of lipoproteins is altered during disease development, similar to other metabolomic parameters [75]. Therefore, lipidomics has been applied for not only gaining insight into lipid metabolism but also for discovering new lipid metabolites associated with both normal and disease states in humans [76]. Many studies have shown that certain lipid species are closely related to disease states. For example, lipidomics using LC–MS on plasma lipids from 1192 individuals from 42 extended Mexican–American families analyzed 319 lipid species and found that increased abundance of two types of diacylglycerols (DG 16:0/22:5 and DG 16:0/22:6) is highly associated with increased systolic, diastolic, and mean arterial pressure as well as prevalent hypertension incidents in Mexican–American populations [39]. Decreased levels of phospholipids in uremic HDL impair the CVD protective effect of HDL [75]. A lipidomics study of plasma from well- and poorly-controlled diabetics versus healthy individuals revealed that not only was HDL-C lower in diabetic plasma, but the percentage of phospholipids and sphingolipids were also significantly higher in diabetic plasma [77]. Quantification of 116 lipid species by a lipidomics analysis in human livers from lean and obese individuals identified that obesity particularly increases oleate-containing diacylglycerols, whereas ceramide and TG remained unchanged [78]. A shotgun lipidomics analysis of human radial, carotid, and femoral endarterectomies found that cholesteryl esters, sphingomyelins, PC, and TGs are the most dominant lipid species [79]. According to a comparison of human endarterectomies and control arteries, CE (CE 18:1) and CE (18:2) were most abundant, followed by PC and sphingomyelin. In addition, more than 40% of CE consisted of CE (18:1) in plasma whereas about 30% of CE was CE (18:1) in the plaques. More diversity of CE species was also found in human plaques compared to plasma, indicating specific lipidome changes in the plaque during atherogenesis [79].

### Lipids as a biomarkers and therapeutic targets

Because of the crucial role of lipids in our body’s metabolism and the observed remodeling of the lipidome in pathophysiological conditions, monitoring levels of individual lipid classes or lipid species can be used to assess disease progression. Lipid abundances can be easily monitored using human biological fluids which include blood, tears, urine, amniotic fluid, and cerebrospinal fluid [79]. In addition, most of these fluids can be collected via non-invasive or minimally invasive procedures, indicating lipid metabolites in biofluids are good candidates for biomarkers [79]. One of the most studied lipid biomarkers in diseases is 24-hydroxycholesterol, which is an oxygenated cholesterol derivative (oxysterol) [73]. Another cholesterol metabolite, 27-hydroxycholesterol promotes atherosclerosis via estrogen receptor alpha-mediated pro-inflammatory events [80]. Plasma 27-hydroxycholesterol is the most commonly found oxysterol in human plasma and it increases with hypercholesterolemia. Its level is also correlated with coronary artery disease [81]. During aging, 27-hydroxycholesterol levels are increased whereas 24-hydroxycholesterol levels remain unchanged [82].

Comprehensive lipidome profiling using global lipidomics has led to the identification of new lipid biomarkers of disease, as well. For example, an untargeted lipidomics analysis of sera collected at 12–14 weeks of pregnancy before the onset of preeclampsia identified 23 potential lipid biomarkers, which can be used to predict the development of preeclampsia during pregnancy [83]. Several lipid mediators like diacylglycerols, oxysterols, ceramides, and free FAs are involved in nonalcoholic fatty liver disease (NAFLD) progression. Lipid profiling on plasma and liver of NAFLD patients identified 48 overlapping lipid species between liver and plasma [84]. This provided candidates for biomarkers that can be used to improve clinical diagnoses without invasive procedures such as biopsies. A comparison of lipidome profiles by shotgun lipidomics using the sera of 30 systemic lupus erythematous (SLE) patients and 30 healthy people identified that all levels of plasmalogen species, which serve as antioxidants, are dramatically reduced in the sera of SLE patients compared to healthy groups [85]. In another study, sphingolipids and inflammatory mediators were found as potential biomarkers for aspirin-exacerbated respiratory disease [86]. Targeted lipidomics discovered that decreased levels of circulating inflammation-resolving lipid mediators (Resolvin D1 and DHA) were associated with acutely symptomatic carotid diseases and strokes [87].

### Lipids as a therapeutic tool

As described above, lipid homeostasis is crucial to prevent metabolic diseases. Since lipids are not encoded by the genome, targeting enzymes that metabolize lipids is one of the ways to control lipid homeostasis. For example, statin drugs block the rate-limiting step of the cholesterol synthesizing enzyme, 3-hydroxy-3-methylglutaryl-coenzyme A (HMG-CoA) reductase, resulting in lower plasma cholesterol level, to treat hypercholesterolemia [88]. In another case, lipids themselves can be used as therapeutic tools because lipids are not merely the byproduct of an enzyme, but they are often bioactive molecules that regulate signaling cascades in cells [44]. In particular, lipids are important mediators of inflammatory responses and the resolution of inflammation [89, 90]. Therefore, lipids can be administered to reduce inflammation, which is involved in nearly all pathologies. Essential FAs (omega-3 and omega-6) administration in inflammatory diseases such as rheumatoid arthritis promoted favorable outcomes [91]. Ocular surface diseases (e.g., chemical and thermal burns, dry eye syndrome, allergic keratoconjunctivitis and infection) are characterized by ocular surface stress and inflammation. These diseases were improved by the lipid-based therapy such as lipid based eye-drops and spray [92]. Administrating omega-3 FAs (EPA and DHA) decreased circulating TG levels by inhibition of lipogenic gene expression, and the anti-inflammatory effects of these PUFA are well established and provide additional benefits [93, 94]. Targeted specialized pro-resolving mediators (SPM) lipidomics on human carotid plaques to compare SPM between stable and vulnerable plaques identified significantly decreased Resolvin D1 (RvD1) in the vulnerable area of plaques [87]. In addition, administration of RvD1 to atherosclerotic mice reduced advanced lesion development and increased plaque stability [87]. In another study, targeted profiling of lipid mediators identified that lipid mediator protectin D1 (PD1) is significantly attenuated in influenza A virus-infected human lung epithelial cells and in mice infected with influenza A [6]. In this study, treating mice with PD1 improved mouse survival and mitigated pathology of influenza-infected mice.

### Current challenges in lipidomics

The field of lipidomics sits in an interesting position as a more recently established sub-discipline of metabolomics, which is beginning to mature simultaneously with the advent of next-generation mass spectrometry instrumentation. Yet despite recent improvements in global lipidomics capabilities driven by MS, a number of significant analytical challenges stand in the way of attaining truly ‘global’ or comprehensive characterization of the lipidome.

A principle challenge to the attainment of truly comprehensive lipidomics analysis is the inability of current methodology to commonly detect more than a fraction of the putative number of lipid species that may comprise the lipidome [27, 95, 96]. Due to current limits of detection, the analysis of some classes of lipids (e.g., eicosanoids, SPMs, and oxysterols) requires separate, ‘targeted’ methodology, often with enrichment of target analytes following extraction of much larger amounts of biological sample than those required for analysis of glycerophospholipids, glycerolipids, sphingolipids, or even non-esterified fatty acids [97, 98]. In some cases, additional derivatization or hydrolysis steps (such as base-catalyzed hydrolysis of cholesteryl esters to enable the analysis of the total sterol pool by reversed-phase LC–MS) are required after the extraction of total lipids from the biological matrix. Moreover, the large number of isomeric/isobaric species of lipids necessitates chromatographic separation in order to accurately identify and quantitate each unique lipid metabolite, precluding the use of ‘shotgun’ approaches to truly achieve full coverage of lipidome. The combination of these issues places limits on the amount of information that may be obtained from any single lipidomics approach. However, some of these challenges may eventually be overcome owing to continuous improvements of mass spectrometry instruments in terms of sensitivity and limits of detection. Coupling such ‘next generation’ MS platforms with more sensitive chromatographic and sample ionization methods, such as nano- or capillary-scale LC–MS and nano-ESI, or gas-phase separation techniques such as ion mobility, may eventually unlock the ability to analyze even the lowest abundance lipid metabolites from biopsy or other ultra-small scale samples.

As discussed earlier, quantification of lipids in global/untargeted lipidomics analyses remains a major challenge as well. Absolute quantification of large numbers of lipid species simultaneously may not be possible due to the limiting number of commercially available synthetic/exogenous lipids that may be spiked into biological samples for use as internal standards. The field of lipidomics has yet to arrive at a general consensus as to how much accuracy is required to quantitate lipids at the level of individual molecular species. Another challenge is the lack of standardization of sample preparation strategies for lipid analysis, which normally includes at least one liquid/liquid extraction step in order to remove lipids from the complex biological matrix. The lack of one universally employed method for sample preparation leaves open the possibility that degradation products or other artifacts resulting from lipid extraction and sample handling may differ across approaches.

## Conclusions

Cumulating studies demonstrate that lipids are actively involved in the onset and progression of metabolic diseases. Recent advances in lipidomics capabilities have made it possible to profile lipidomes of cellular organelles, cells, tissues, organs, or whole organisms, in various pathophysiological conditions. Although several challenges remain for current lipidomics approaches, lipidomics has already begun to contribute to our understanding of the dynamic and key roles of lipid metabolism in health and disease while enabling the identification of disease biomarkers, thereby facilitating precise diagnosis of diseases and opening the door to individualized medicine.
